# Review on the Antibacterial Mechanism of Plant-Derived Compounds against Multidrug-Resistant Bacteria (MDR)

**DOI:** 10.1155/2021/3663315

**Published:** 2021-08-16

**Authors:** Najwan Jubair, Mogana Rajagopal, Sasikala Chinnappan, Norhayati Binti Abdullah, Ayesha Fatima

**Affiliations:** ^1^Faculty of Pharmaceutical Sciences, UCSI University, Kuala Lumpur 56000, Malaysia; ^2^Forest Research Institute Malaysia (FRIM), Kuala Lumpur 52109, Selangor, Malaysia; ^3^Beykoz Institute of Life Sciences and Biotechnology, Bezmialem Vakif University, Istanbul, Turkey

## Abstract

Microbial resistance has progressed rapidly and is becoming the leading cause of death globally. The spread of antibiotic-resistant microorganisms has been a significant threat to the successful therapy against microbial infections. Scientists have become more concerned about the possibility of a return to the pre-antibiotic era. Thus, searching for alternatives to fight microorganisms has become a necessity. Some bacteria are naturally resistant to antibiotics, while others acquire resistance mainly by the misuse of antibiotics and the emergence of new resistant variants through mutation. Since ancient times, plants represent the leading source of drugs and alternative medicine for fighting against diseases. Plants are rich sources of valuable secondary metabolites, such as alkaloids, quinones, tannins, terpenoids, flavonoids, and polyphenols. Many studies focus on plant secondary metabolites as a potential source for antibiotic discovery. They have the required structural properties and can act by different mechanisms. This review analyses the antibiotic resistance strategies produced by multidrug-resistant bacteria and explores the phytochemicals from different classes with documented antimicrobial action against resistant bacteria, either alone or in combination with traditional antibiotics.

## 1. Introduction

Bacterial infection is considered as one of the significant contributors to human illness in developed and developing countries [1]. Pathogens evolve with time and gain resistance to formerly discovered antibiotics. Among two million people infected each year worldwide with various types of bacteria, 700 thousands of them died because of bacterial resistance [2, 3]. For instance, methicillin-resistant *Staphylococcus aureus* accounts for 50,000 deaths annually in the United States and Europe [4]. Simultaneously, antibiotic-resistant *Mycobacterium tuberculosis* infected about 480,000 people in underdeveloped countries during 2013 [4].

According to the CDC's antibiotic-resistance threat report in 2019, over 2.8 million antibiotic-resistance infections were recorded in the United States [5]. Carbapenem-resistant *Acinetobacter* seems to be the top, urgent threat, with 700 estimated deaths in the United States in 2017 [5]. This number is estimated to increase to ten million cases by 2050 due to lack of response to medication despite all efforts and costs to overcome this issue, which is estimated to be $100.2 trillion by 2050 [6]. Some bacteria are innately resistant to antibiotics, while others acquired resistance with time. According to the European Center for Disease Prevention and Control (ECDC), Center for Disease Prevention and Control (CDC), and Infectious Diseases Society of America (IDSA) [7], acquired bacterial resistance can be categorized into three main classes: extensive-drug resistance (XDR), pan drug resistance (PDR), and multidrug resistance (MDR) [8].

Extensive-drug resistance (XDR) is defined as nonsusceptibility to at least one antibiotic in all but two or fewer antimicrobial classes, which means that bacterial strains remain sensitive to only one or two antibiotic categories [9]. An example of this class is XDR-*M*. *tuberculosis*, which resists isoniazid, rifampicin, fluoroquinolones, and at least one agent of the three second-line treatments (amikacin, kanamycin, or capreomycin) [10]. For pan drug resistance (PDR), the name derives from the Greek word “pan,” which means “all,” referring to bacterial resistance to all commercially available or routinely tested antimicrobial agents [8].

Multidrug-resistant microorganisms (MDR) assign bacteria resistant to a minimum of one agent in three or more antimicrobial classes [7]. The most common types of MDR bacteria are methicillin-resistant *S. aureus* (MRSA), vancomycin-resistant *Enterococci* (VRE), carbapenem-resistant *A. baumannii* (CRAB), multidrug-resistant *P. aeruginosa*, *Enterobacteriaceae* that produces extended-spectrum *β*-lactamases, and carbapenem-resistant *Enterobacteriaceae* (CRE) [11]. These resistant strains are considered a major threat to human health as the outcome associated with MDR bacteria is worse than patients infected with more susceptible organisms, and the rising cost associated with these infections [11].

Consequently, scientists have become more concerned about the efficacy of current antibiotics against various types of infectious diseases. Many studies concentrated on finding new alternative medication as a way to overcome resistant problems. Nature represents a potential source for drug discovery. For decades, plants' phytochemicals attracted scientists as they are numerous in structure, have fewer side effects, and are more acceptable by people [12, 13]. Most of these phytochemicals have antimicrobial activity [12, 14]. Approximately, 25% of the current pharmacopeia is from plant-derived compounds [15].

About 80% of the developing countries depend on plant-derived medicines as their first-line treatment [16]. Moreover, plants remain the core of several medical practices such as Ayurveda (traditional Indian medicinal systems) and Traditional Chinese medicine (TCM) [17]. For instance, *Oxalis corniculata* extract has intense antimicrobial activity against *E. coli, MDR-Salmonella typhi, K. pneumoniae*, and *Citrobacter koseri* (inhibition zones are 17 mm, 13 mm, 16 mm, 11 mm, and 12 mm, respectively) [18].

Five local plants that have been used by indigenous people in Iraq were tested for their antimicrobial effect toward *S. epidermidis* and *K. pneumoniae* [19]. It was shown that the type of solvents and the extract concentration markedly affect their activity, with *Punica granatum* L. being the most active extract regardless of the solvent type [19]. *Ludwigia parviflora* Roxb. plant has been used traditionally in India for wound dressing and as a remedy for dysentery [20]. The ethanolic fruit extract of this plant showed significant antibacterial activity toward *E. coli, B. subtilis, S. typhi,* and *S. pyogenes* in a study that emphasizes the use of this plant traditionally to treat various diseases [20]. The present review focused on describing the bacterial mechanism of resistance, and providing an updated review of plant phytochemicals' antibacterial mechanism against multidrug-resistant bacteria.

## 2. Mechanism of Bacterial Resistance

About 20,000 resistant genes have been identified from bacteria, and various mechanisms have been highlighted to explain bacterial resistance to traditional antimicrobial agents [21]. The first case of antibiotic resistance was detected during the 1950s with *Salmonella, Shigella,* and *E. coli* species [22]. However, it took two decades to recognize this rising problem, as in the 1970s, several cases were reported with resistance to penicillin, tetracyclines, and chloramphenicol [22]. The proposed mechanisms for bacterial resistance were rarely tested on a clinical basis [23]. It is ambiguous whether each microorganism has its mechanism, or there is a similar pathway shared by several bacteria to produce resistance [23].

Some bacteria are naturally resistant to antibiotics, while others acquire resistance mainly through the misuse of antibiotics and the emergence of new resistant strains [24]. The mechanism of antibacterial resistance ranges from accelerating antibiotics efflux through bacterial efflux pumps so as to decrease the time required for medication to diffuse inside bacteria [25], alteration of bacterial porins' structure which decreases bacterial permeability to antibiotic influx [26], destruction of antibacterial agents by hydrolytic enzymes [25], to alteration of binding sites for antibiotics [27]. For bacteria, they may fight antibiotics by one mechanism or by combining more than one to produce their resistance [25]. Understanding resistance mechanism is essential to identify the possible target for effective medication in the future. Each of the mechanisms mentioned above will be discussed in detail as follows.

### 2.1. Efflux Pump

To produce antibacterial activity, the antibiotic needs to enter the bacterial cell at adequate concentration and stay for a considerable time to perform its action [25]. Many MDR bacteria counter antibiotics by the efflux pump mechanism. Efflux pumps are proteinaceous transporters located in the cytoplasmic membrane that function as regulators of the bacterial internal environment [28]. They aid bacteria in removing toxins as well as antibiotics. For the first time, scientists identified the efflux pump as the cause of *E. coli* resistance to tetracycline [29]. However, it was recently considered a major cause of resistance [28].

**Figure 1:**
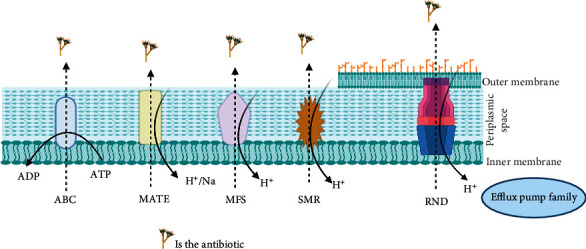
Schematic representation showing the major types of efflux pump families present in bacteria. Presented are resistance-nodulation family (RND) which is specific for Gram-negative bacteria; small multidrug resistance (SMR) locates in the inner membrane; major facilitator superfamily (MFS), multidrug and toxin extrusion (MATE), and adenosine triphosphate-binding cassette (ABC) superfamily are located in the inner membrane. All efflux pumps regulate toxins and antibiotics transports in energy-dependent manner.

Based on sequence similarities, source of energy, substrate binding, and the number of components, bacterial efflux transporters are classified into five prominent families [28]; Resistance-nodulation division (RND), which is a specific group for Gram-negative bacteria [30], adenosine triphosphate (ATP)-binding cassette superfamily (ABC) [31], multidrug and toxic compound extrusion (MATE) [32], major facilitator superfamily (MFS) [33], and small multidrug resistance family (SMR) [34].  Figure 1 is adopted from Blanco et al., presenting the major efflux transporters in bacteria [28]. ABC, MATE, MFS, and SMR distribute to a large degree in Gram-positive and Gram-negative bacteria [28].

Through efflux pumps, bacteria extrude antimicrobial agents faster than usual. Thus, the agent's time to reach the target site at a considerable concentration will decrease [25]. Bacterial efflux pumps are encoded by genes located either in mobile genetic elements (MGEs), representing genetic materials like plasmids and transposons found in all organisms, including humans or in chromosomes [35]. Recently, about 20 genes encoded in bacterial efflux have been identified, most of them located in MGEs and modulate bacterial resistance of most Gram-positive bacteria [28]. Various MDR efflux pumps in *Enterobacteriaceae* and *P. aeruginosa* belong to the RND family, and effectively extrude tetracyclines [36].

Due to RND pumps, resistance also occurs in many antibiotics like chloramphenicol, beta-lactams, fluoroquinolones, and fusidic acid [28]. *S. pyogenes*, *S. pneumoniae*, *Streptococci*, and some Gram-positive bacteria resist macrolides by efflux pumps mainly through the ABC family present in the chromosome [37]. This mechanism of resistance was initially recognized in *S. epidermidis* [37]. Antibiotic resistance through efflux pump mechanism also manifests in *A. baumannii* by which AdeABC and RND efflux pumps release antibiotics like aminoglycosides, tigecycline, lactams, chloramphenicol, erythromycin, and tetracycline from the cell [38], which results in a reduction of drug accumulation at the target site and increases the minimum inhibitory concentration of antibiotics making them ineffective [38].

### 2.2. Alteration of Membrane Permeability

**Figure 2:**
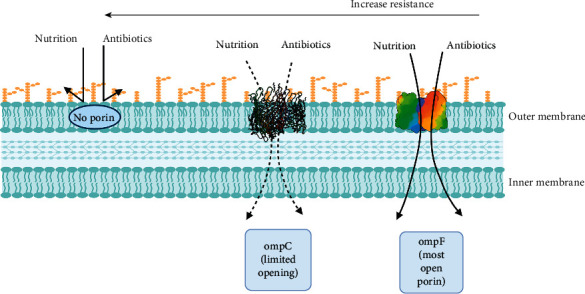
Effect of porins in membrane permeability of *E. coli*.

Gram-negative bacteria are characterized by the presence of an outer membrane (OM), which functions as an additional protection layer against harmful compounds [39]. Porins are proteins present in the bacterial outer membrane that can form water-filled holes controlling the passage of several materials and nutrients through this membrane [25]. They represent one of the possible targets for antibacterial agents [25]. Porins were first identified in *E. coli* (Figure 2 adopted from Katherine Phan and Thomas Ferenci illustration) [40]. They can be classified based on activity into specific and nonspecific [41]. Specific porins are more selective for certain compounds such as Lam B, and selectively uptake maltose, maltodextrin, and Fep A specific for iron complex [42]. In contrast, nonspecific or general porins are involved in membrane permeability and linked to bacterial resistance to the antimicrobial agents [42].

Based on structure, porins are divided into monomeric, dimeric, and trimeric [39]. Besides, they can be classified according to their role in antibiotic transport and membrane integrity into three groups; specific porins for antibiotic transport (LamB, YddB), porin specific for membrane integrity (OmpA), and nonspecific porins act on both antibiotic transport and membrane safety (OmpC, OmpF) [39]. Alteration of the porin structure is associated with an increase in bacterial resistance to antibiotics [25]. For example, OmpF porin is responsible for the transportation of several antibiotics such as *β*-lactams and fluoroquinolones.

Mutation in this porin renders resistance in *E. coli* [43], *K. pneumoniae* [44], and *P. aeruginosa* [45]. *A. baumannii* resists carbapenem by reducing porin expressions such as Caro and Omp 22–33. Besides, it changes the outer membrane integrity to acquire colistin resistance [46]. The mechanism by which *P. aeruginosa* resists various types of antibiotics is classified into intrinsic, acquired, and adaptive [47]. Alteration of the outer-membrane main component, the liposaccharides (LPs), is an example of intrinsic-type resistance by which *P. aeruginosa* fights antibiotics like *β*-lactams, quinolones, and aminoglycosides [47].

### 2.3. Destruction of Antibiotics

**Figure 3:**
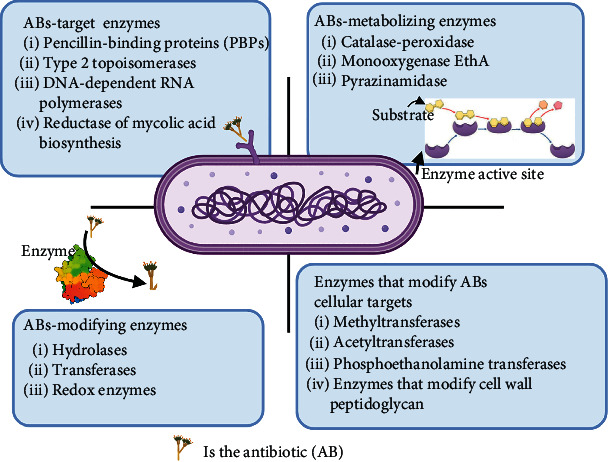
Bacterial enzymes that are involved in various mechanisms of microbial resistance.

Bacterial enzymes (Figure 3, adopted from Egorov et al. [48] with slight modifications) are a group of enzymes associated with the biosynthesis of the bacterial cell wall, nucleic acids, and metabolites synthesis. Some antimicrobial agents directly target these enzymes as part of their mechanism. Thus, bacteria might resist antibiotics through structural changes or modification of the structural elements affected by antibiotics: for example, modification of ribosomes by methyltransferases [49].

Both Gram-positive and Gram-negative bacteria release enzymes to hydrolyze or modify antibiotic molecules, rendering them inactive [25]. Most antibiotics inactivated by this mechanism act through inhibition of bacterial protein synthesis [50]. Three types of enzymes are known to cause antibiotic resistance [51]:

1. *β*-Lactamase enzymes hydrolyze the lactam ring in penicillin, cephalosporines, and carbapenems [52]. About 300 types of *β*-lactamases are identified based on structural and functional properties.
2. Chloramphenicol acetyltransferase acetylates the hydroxyl group in chloramphenicol, making it unable to bind correctly to the target site. *Haemophilus influenza*, Gram-negative *Bacilli*, *Enterobacteriaceae*, *Acinetobacter*, and *P. aeruginosa,* resist chloramphenicol by using this mechanism [53].
3. Aminoglycoside-modifying enzyme, *S. aureus*, *S. pneumoniae*, and *E. faecalis* fight antibiotics by producing enzymes that modify drug molecule and decrease its affinity for binding to 30S subunits [54].

### 2.4. Alteration of the Binding Site

Bacteria may resist antibiotics by remodeling the bacterial target site in a way that decreases the binding affinity of antimicrobial agents [25]. Erythromycin binding interaction is markedly reduced by methylation of the target site, a peptidyl transferase enzyme [55]. Besides, *Staphylococci* acquired resistance to methicillin and oxacillin through mutational changes in penicillin-binding proteins (PBPs) [56]. Vancomycin-resistant *Enterococci* (VRE) counter antibiotic action by changing the amide linkage of the targeted enzyme into ester linkage, resulting in 1000 times less antibiotic binding affinity [57].

*Helicobacter pylorus* is a Gram-negative bacterium responsible for gastric infections such as gastritis, gastric ulcers, and gastric cancer [58]*. H. pylori* resist the first-line treatment agent “clarithromycin” via different mutations in the domain V of the 23S rRNA gene like A2142G, A2142C, or A2143G. These mutations decrease the affinity of bacteria to the antimicrobial agents [46]. Gram-positive bacteria alter antibiotic-binding sites making them ineffective, like modifying ribosomal targets to acquire resistance to macrolides, lincosamides, streptogramins, tetracyclines, and aminoglycosides [59].

**Figure 4:**
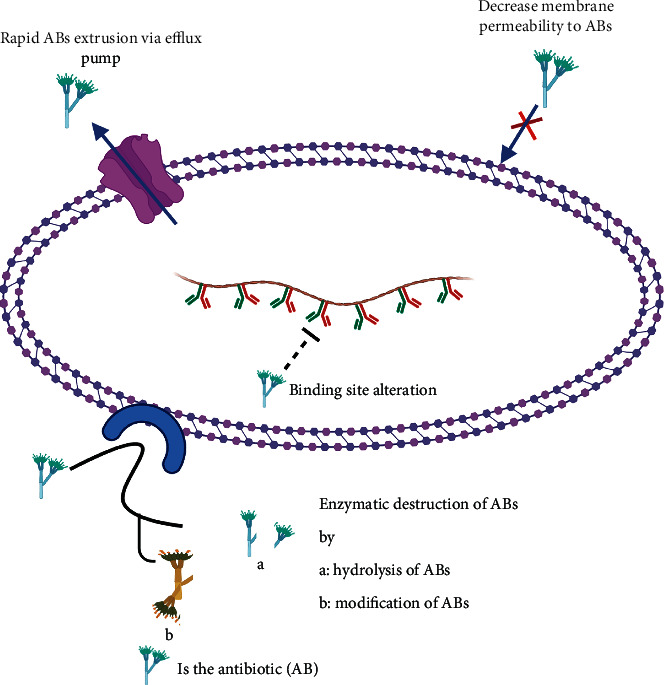
Schematic representation of antibiotic resistance.

Figure 4, adapted from Giannamaria Annunziato, represents a schematic illustration of antibiotic resistance mechanisms.  Table 1 shows the mechanism of resistance and the type of antibiotics affected by common multidrug-resistant bacteria.

## 3. Plant-Derived Compounds

Since ancient times, herbal medicine was considered a cornerstone for treating various medical conditions such as cold, diarrhea, dental disease, and labor pain [66]. They represent patients' first treatment choice as they are associated with fewer side effects, and are cheap and affordable, especially in underdeveloped countries [67]. Even though approximately 17,000 plant species have been identified, only 3,000 species are in medical use [68]. However, according to the World Health Organization, 80% of people rely on folk medicine for their healthcare demands [66]. As a result, the search for medicinal plants with promising activity gains additional value. Plants are extensively investigated as a leading source for the synthesis of new therapeutic agents, and the possibility of using crude extracts in the treatment of different diseases [69].

Plants produce two types of metabolites: primary, which is essential for plant survival, and secondary, which resulted in response to plant interaction with the environment [70]. Primary metabolites are products of glycolysis, shikimate pathway, and tricarboxylic acid cycle, which aid in nutrition and reproduction [71]. In addition, they act as a precursor for thousands of secondary metabolites that are produced at different steps in primary metabolic pathways [72], which provokes the role of enzymatic activities against primary metabolites in the production of new compounds that support plant adaptation against biotic (e.g., bacteria, fungi, insect, and disease) and abiotic (e.g., injury, temperature, and moisture) stress [70].

Plant-derived compounds are secondary products of plant metabolism present in different parts of the plant, such as root, leaves, bark, flowers, seeds, and characterized by wide structural diversity [73, 74]. For decades, plant-derived compounds acquired extra attention in the drug industry to enhance existing antibiotics' biological activity or as a potential source for new antimicrobial agents active against several pathogens, including MDR bacteria (13, 25, 71). Based on their biosynthetic origin, plant phytochemicals can be divided into terpenoids, polyketides, phenylpropanoids, and alkaloids [71]. This review demonstrates the update in plant phytochemicals' antimicrobial activity along with their antibacterial mechanism of action against various MDR bacteria. In this review, plant-derived compounds were sectioned into alkaloids, polyphenols, terpenoids, sulfur-containing compounds, and coumarins according to their chemical structures.  Table 2 lists plants that reported antimicrobial activity against MDR bacteria from 2015 to 2021.

### 3.1. Alkaloids

Alkaloids are organic nitrogenous compounds with substantial structural diversity [25]. The name “alkaloids” was introduced in 1819 by German chemist Carl Friedrich Wilhelm Meissner to refer to natural products of plant origin that showed basic properties similar to alkalis, but Friedrich Wilhelm Adam Sertürner is best known for his discovery of morphine in 1804; Sertürner isolated the first alkaloids [99]. These molecules' biosynthetic origin involves various organisms like bacteria, fungi, plants, and animals [100].

According to their biological sources, alkaloids may be classified into natural, semi-synthetic, and synthetic alkaloids, or based on their chemical structure into typical alkaloids with heterocyclic ring, and atypical alkaloid nonheterocyclic. This classification is further split into several classes: tropanes, indole, purines, imidazole, pyrrolidine, pyrrolizidine, isoquinoline, piperidine, and quinolizidine [100]. More than 18,000 types of alkaloids were identified from different sources [101].

The antimicrobial activity of alkaloids has been documented since the 1940s [102]. It has believed that the nitrogen atom is responsible for the bioactivity of this class [103]. Moreover, the antibacterial mechanism of alkaloidal phytochemicals against various infectious diseases is through efflux pump inhibition [25].  Table 3 shows the chemical structures of selected alkaloidal compounds with antimicrobial activity against MDR bacteria. For instance, sanguinarine **(1)** is an isoquinoline alkaloid present in various plants like *Sanguinaria canadensis* L., *Fumaria officinalis* L., *Bocconia frutescens* L., *Chelidonium majus* L., and *Macleaya cordata* (Willd.) R. Br. [115]. This compound exhibits broad pharmacological activity.

Concerning antimicrobial activity, sanguinarine isolated from root and aerial parts of *Chelidonium majus* L. exhibited a significant effect toward *S. aureus* strains with MIC 1.9 mg/L. It is suggested that iminium bond, methoxy substitution, and the charge on quaternary nitrogen atom are responsible for this antibacterial action [104]. In a study, sanguinarine prevents bacterial cell division in *E. coli* by inhibiting cytokinetic Z ring formation [105]. This alkaloid effectively suppresses *MRSA* growth by weakening the bacterial cytoplasmic membrane, according to another study. When *MRSA* is exposed to sanguinarine, it will produce autolytic enzymes to induce cell lysis [106]. In a recent study, sanguinarine exhibited potent inhibitory effects (MIC 7.8 *μ*g/mL) on *Providenica rettgeri*–resistant isolates [116]. According to confocal laser scanning microscopy (CLSM), field emission scanning electron microscopy (FESEM), and crystal violet staining results, sanguinarine affected bacterial biofilm formation and resulted in a decrease in the intracellular ATP concentration [116].

The synergistic activity of sanguinarine was also documented. A study on the effect of sanguinarine, EDTA, and vancomycin on 34 strains of Gram-positive and Gram-negative bacteria was conducted. In the previous study, sanguinarine alone showed good activity against all strains with MIC 0.6–128 *μ*g/mL. Time kill study revealed that this alkaloid's bactericidal effect appeared after 4–6 h. EDTA had a bacteriostatic effect, and the majority of Gram-negative bacteria resist vancomycin. However, this combination increases the sensitivity of resistant bacteria to vancomycin [117].

Similarly, a combination with streptomycin was studied. MIC and time-kill assay showed that sanguinarine, EDTA, and streptomycin had significant activity against *K. pneumoniae* and *E. coli* but were inactive against *MSRA* and *S. aureus* [118]. These combinations proposed a possible strategy to overcome resistance. Another isoquinoline alkaloid is berberine [2], which is present in roots, stem-bark, and rhizome of various herbs such as *Hydrastis canadensis* L., *Berberis aristata* DC., *Coptis chinensis* Franch., *Coptis japonica* (Thunb.) Makino., and *Phellodendron amurense* Rupr. [119]. It has been known for a thousand years in China for its antimicrobial activity [119]. Interaction with bacterial DNA, inhibition of bacterial protein FtsZ responsible for cell division, and enzyme targeting are possible mechanisms for this antibacterial action [25].

Besides, berberine has a synergistic effect by enhancing the bacterial inhibition of some antibiotics [107]. In a previous study, a combination of berberine with ten different antibiotics was assessed for its antimicrobial activity against 14 *Staphylococcus* isolates. MIC values ranged from 16–512 *μ*g/mL. A 5 mm increase in the inhibition zone was observed with seven isolates, and berberine's combination with linezolid, cefoxitin, and erythromycin exhibited profound improvement in the antibacterial activity [107]. However, the mechanism underlying such effect is poorly understood.

However, in a recent study, it was noticed that berberine affects membrane integrity by altering the fatty acids contents in both saturated and unsaturated fatty acids, and disrupting *MRSA* cell surface in a dose-dependent manner [120]. K^+^ leakage and periplasmic alkaline phosphatase concentration had been assessed as an indication of membrane permeability changes. Furthermore, morphological alterations were detected in scanning electron microscopy (SEM) and transmission electron microscopy (TEM). Berberine-treated cells showed a nonspherical shape, and some of them have a doughnut-shaped structure without a central hole. In a recent report, berberine displayed a bacteriostatic effect against *MRSA* [108]. Molecular dynamics simulation data indicated that berberine could bind to the phenyl ring of Phe19 in PSM*α*2 via hydrophobic interaction. In addition, it can inhibit *MRSA* biofilm formation by influencing PSMs' aggregation into amyloid fibrils, which explains berberine's synergistic effect [108].

Solasodine **(3)** and Tomatidine **(4)** are essential glycoalkaloid metabolites isolated from the *Solanaceae* genus [121]. These molecules are well-known for decades for their biological activity and as starting compounds for synthesizing steroid drugs [122]. According to a recent study, genomic analysis was performed to identify the cause of resistance in *MRSA*. It was shown that modification of genetic sequences in bacterial ATP synthase confronted resistance to antibiotics [109]. Tomatidine appears to target this enzyme; thus, it could be considered a lead source to develop new antibiotics to combat bacterial resistance [109]. Tomatidine synergy was also evaluated against *S. aureus*, *E. faecalis*, *P. aeruginosa,* and *E. coli* using checkboard method and FIC indices. The results indicated that tomatidine has no antibacterial activity toward previously mentioned strains, but it inhibited *S. aureus* and *P. aeruginosa* growth combined with gentamycin or cefepime. A similar effect was recognized when *P. aeruginosa* and *E. faecalis* were exposed to tomatidine with ciprofloxacin or ampicillin [110].

Conessine **(5)** is another steroidal alkaloid isolated from the stem-bark of different species belonging to the *Apocynaceae* family, such as *Holarrhena antidysenterica* (Roth) Wall. ex A.DC. [111]. This plant's extract has been used in Thai folk medicine as antidiarrheal, antibacterial, and astringent. These effects can be attributed to steroidal alkaloid contents, mainly conessine [123]. Available studies indicated overexpression of efflux pumps, especially Mex-oprM confer *P. aeruginosa* resistance to Antibiotics [124, 125]. Thus, using EPI and existing antibiotics represent one of the strategies to restore antibiotic activity [126]. Coadministration of conessine with levofloxacin was tested against *P. aeruginosa* clinical isolates with a mutation in MexAB-OprM, MexcD-OprJ, and MeXEF-OprN efflux pumps [111]. MIC and time-kill study evaluated the efficacy of using levofloxacin alone and with alkaloid combination. The result showed that conessine successfully retrieved levofloxacin efficacy against *P. aeruginosa* strain with efflux pumps mediated resistance [111]. In an analogous manner, conessine has a synergistic effect when combined with novobiocin or rifampicin in treating extensively drug-resistant *A. baumannii* infection [112].

Anti-malarial compounds such as quinoline alkaloid evocarpine **(6)** and quinolines like dictamnine and masculine are known to have noticeable antimicrobial activity [113, 127]. Evocarpine exhibited higher antibacterial activity than isoniazid in the treatment of *M. tuberculosis* infection. This effect relates to the inhibition of enzymes required for bacterial cell wall synthesis [113]. Coadministration of ergot alkaloid chanoclavine **(7)** with tetracycline markedly suppresses resistant *E. coli* growth [114]. Although chanoclavine has no antibacterial activity alone, with tetracycline it shows considerable synergy. The mechanism underlying this action is due to chanoclavine-mediated inhibition of bacterial efflux pump, which is ATPase dependent. *In silico* docking showed the ability of chanoclavine to bind with various proteins involved in drug resistance [114]. Alkaloidal antibacterial activity of two plant leaves extracts (*Callistemon citrinus* (Curtis) Skeels. and *Vernonia adoensis*) were tested against MDR *S. aureus* and *P. aeruginosa*. *Callistemon citrinus* (Curtis) Skeels. showed potent antibacterial activity with MIC = 0.0025 mg/mL, and efflux pump inhibition caused by rhodamine 6G accumulation of 121% compared to control [128].

Tetrahydrosecamine (piperidine alkaloid) and Strictanol (indole alkaloid) isolated from the leaves of *Rhazya stricta* Decne. exhibited significant antimicrobial activity toward *MRSA*, *E. coli,* and *P. aeruginosa* through disruption of the bacterial cell membrane [74]. Similarly, two isoquinoline alkaloids isolated for the first time from *Zanthoxulum tingoassuiba* root bark, dihydrocheleryhtrine, and N-methylcanadine showed good antibacterial activity (MIC 60 *μ*g/mL) against *MRSA* [129]. Four papaver species from the *Papaveraceae* family grown naturally in Iran were tested for the antimicrobial activity for both Gram-positive and Gram-negative pathogens. Benzylisoquinoline alkaloids were identified from *Papaver macrostomum* Boiss. & A.Huet., *Roemeria refracta* DC., *Papaver somniferum* L., and *Glaucium grandiflorum* tissues. The alkaloidal extracts of these plants were tested against *P. aeruginosa*, *S. aureus,* and *K. pneumoniae*. All extracts displayed considerable inhibitory effects for Gram-positive pathogens more than Gram-negative bacteria. *Roemeria refracta* DC. alkaloid has a stronger inhibitory effect toward *S. aureus* than other species (MIC 0.065 *μ*g/mL) [130].

In the Middle East, *Salvadora persica* is traditionally used as a chewable stick to ensure oral hygiene. Methanolic and aqueous extract of this plant were studied for their antimicrobial activity against MDR *S. aureus* and *P. aeruginosa*. Alkaloids, saponin, flavonoids, terpenoids, and volatile oils were isolated from *S. persica* extract [131]. The aqueous extract showed better inhibition with MIC equal to 2.49 mg/mL for *S. aureus* and 7.34 mg/mL for *P. aeruginosa*. However, the specific compound responsible for this activity was not identified yet [60].

*Black zira* essential oils exhibited bacteriostatic and bactericidal activity toward several pathogens, including *P. aeruginosa* and *E. coli*, with MIC ranging from 1 mg/mL to 8 mg/mL and MBC values ranging between 1 and 16 mg/mL. Phytochemical analysis revealed the presence of alkaloids, saponin, flavonoids, tannin, and phenols [131]. The antimicrobial activity of leaves' extracts of *Carica papaya* L., *Datura stramonium* L., and fruit extract of *Piper nigrum* L. was tested against Gram-positive and Gram-negative bacteria including MDR *S. aureus*, *P. aeruginosa*, *A. baumannii,* and *E. coli*. All extracts showed better activity against Gram-positive bacteria. However, *Piper nigrum* extract had no activity toward MDR. Ethanolic extracts of *Carica papaya* L. and *Datura stramonium* L. displayed larger zone of inhibition than methanol extracts. The phytochemical screening for both plants indicated the presence of alkaloids, steroids, glycosides, phenols, saponin, and carotenoids with tannin present only in *Datura* extract [132]. Several alkaloids were identified in a southeast Asian plant, *Piper sarmentosum* Roxb.; some are present in roots and stems like langkamide, piplartine, and trimethoxycinnamic acid, while amide alkaloid compounds were isolated from plant leaves. Methanolic extracts of the leaves exhibited good antibacterial activity, including MDR pathogens such as *MRSA* and *E. coli* with MIC 100 mg/mL [133].

### 3.2. Polyphenols

Polyphenols are a large group of secondary plant metabolites with phenolic pharmacophore [134]. More than 8000 polyphenols were identified with biological activity mainly as antioxidants that protect plant and human cells from free radical damage [135]. They are classified into four categories according to the number of phenolic groups and the structural elements as follows: (1) flavonoid, which further split into flavones, isoflavones, flavonols, flavanones, chalcones, and catechins; (2) stilbenes with resveratrol as the main compound; (3) lignans and finally (4) phenolic acids like hydroxybenzoic acid and hydroxycinnamic acid [136].

**Figure 5:**
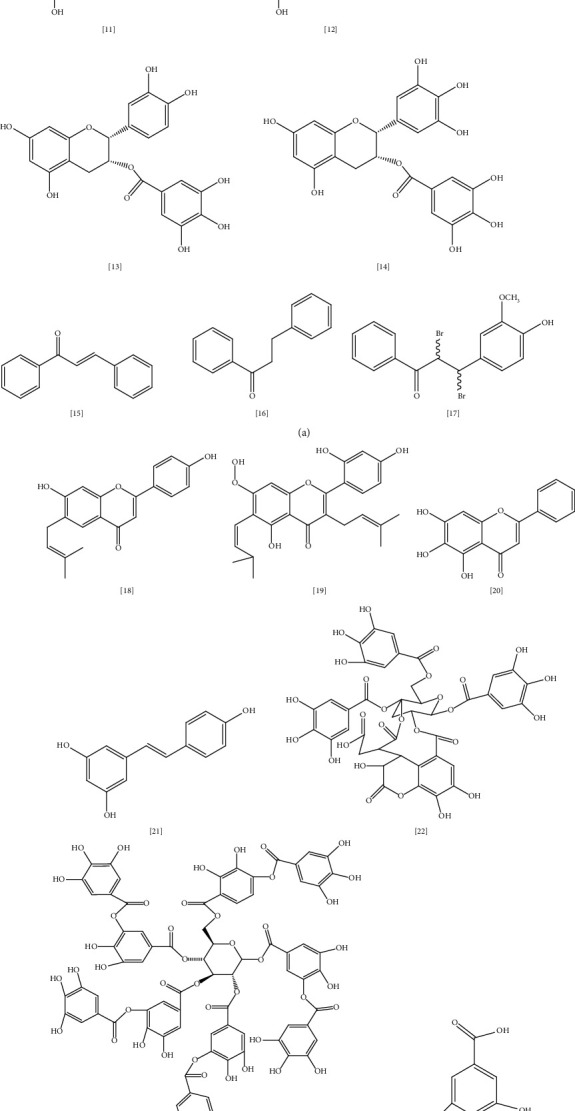
Chemical structures of selected alkaloids with antimicrobial activity against MDR bacteria.

Besides polyphenols' central role as an antioxidant, antiinflammatory, antiallergic, anticancer, antihypertensive, and antimicrobial activities were also documented [137].  Figure 5 illustrates the chemical structures of some polyphenols with documented antimicrobial activity against MDR. Although the exact mechanism for polyphenols' antimicrobial action is not fully understood, several studies suggested that at the cellular level, polyphenols bind to bacterial enzymes via a hydrogen bond, inducing several modifications in cell membrane permeability and cell wall integrity [138].

One of the most abundant flavonoids present in food are flavanols, which have potent antimicrobial activity against Gram-positive and Gram-negative pathogens, including resistant strains [139]. Examples of this group include quercetin **(8)** and kaempferol **(9)**. In one study, a combination of quercetin with amoxicillin exhibited synergistic activity against *S. epidermidis* isolates that are resistant to amoxicillin alone; however, the activity was markedly reserved when quercetin was added [140]. According to the study, quercetin inhibited beta-lactamase and bacterial peptidoglycan synthesis, as well as increased cell membrane permeability and decreased fatty acids in bacterial cells [140]. Similarly, quercetin's synergy with levofloxacin, ceftriaxone, gentamycin, tobramycin, and amikacin has been tested against *P. aeruginosa*. The biofilm formation and biofilm cell viability had been markedly affected by this combination with ≥80% inhibition. Bactericidal effect was significant as well with (68 to 85%) cell killing [141]. In another study, a combination of low-dose quercetin and tetracycline exhibited bactericidal action against antibiotic-resistant *E. coli* in a mouse-infected model. As a mechanism, quercetin disrupted the bacterial cell envelope, resulting in increased cell permeability and lysis [142]. These results encourage more focus on combining antibiotics with phytochemicals as a new approach to combat bacterial resistance.

Kaempferol and its derivatives isolated from *Bryophyllum pinnatum* displayed significant antibacterial activity against several pathogens, including antibiotic-resistant *S. aureus* and *P. aeruginosa* [143]. For the last decade, kaempferol acquired extra attention after its activity against *MRSA* had been proven [144]. This action's mechanism is thought to be through kaempferol-mediated inhibition of NorA efflux pump [145]. Om Prakash et al. studied the effect of quercetin and kaempferol combination with regular antibiotics in the antibacterial activity of these antibiotics against *MRSA* [146]. According to the study, antibiotics like cefixime, ceftriaxone, ciprofloxacin, cephradine, methicillin, ampicillin, and amoxicillin, when mixed with quercetin, kaempferol, or quercetin + kaempferol, revealed remarkable MIC values against *MRSA,* which is usually resistant to these antibiotics when administered alone. However, a combination of classical antibiotics with these flavonoids restores their antibacterial activity [146].

*Camellia sinensis* (green tea) is a plant well-known for its antioxidant and antimicrobial properties against various pathogens, including resistant *S. aureus* and *P. aeruginosa* [147]. In a previous study, green tea extract was reported to be active against 11 isolates of resistant *E. coli* in patients who suffered from UTI infections in Nepal (MIC 600 *μ*g/mL) [148]. Catechin **(10)** is a polyphenol abundant in green tea and accountable for the plant's activity. Four forms of catechin are present in green tea; epicatechin (EC) **(11)**, epigallocatechin (EGC) **(12)**, EC-3-gallate (ECG) **(13)**, and EGC-3-gallate (EGCG) **(14)** [147]. Several studies indicated the pharmacological importance of catechin phytochemical [147, 149, 150]. In a study, catechin showed metal-chelating properties making it more effective as an antioxidant than vitamin C [149]. Moreover, green tea extract was shown to be active against *P. aeruginosa* in several studies [147]. Catechin gallates effectively reverse *MRSA* resistance through NorA efflux pump inhibition. It has two different binding sites in the NorA substrate and causes EPI at high concentrations [151].

Chalcones are considered important secondary metabolites and precursors of flavonoids and isoflavonoids in plants [152] that produce their antimicrobial activities through NorA efflux pump inhibition [153]. Chalcone **(15)** and dihydrochalcone **(16)** isolated from the ethanolic root extract of *Uvaria chamae* P. Beauv. were assessed for the first time by Koudokpon et al. for their antibacterial activity against Gram-positive MDR pathogens [154]. Ten chalcones were identified by ion mobility MS analysis with significant inhibition of *VRE*, *MRSA,* and *S. aureus* growth [154]. Some research documented a considerable increase in antibacterial activity of chalcones in combination with antibiotics. An example is a chalcone isolated from traditional African and American herb roots, *Sophora flavescens* Aiton. [155]. Following the previously mentioned study, 7,9,2′,4′-tetrahydroxy-8-isopentenyl-5-methoxychalcone was found to be active against *MRSA* and *VRE* alone or in combination with ampicillin or gentamycin [155]. 4′,6′-Dihydroxy-3′,5′-dimethyl-2′-methoxychalcone from *Dalea versicolor* Zucc. was found to enhance erythromycin activity by reducing MIC from 0.4–0.1 *μ*g/mL in another study [153].

Several studies reported that synthesized flavonoids' derivatives could be more effective as antibacterial agents than natural flavonoids [156–158]. Seventeen chalcone derivatives were synthesized and tested for their antimicrobial activity against eleven bacteria strains, including *S. aureus* and *E. faecalis* [158]. All compounds significantly inhibited bacterial growth, with one compound (dibromo derivative) **(17)** showing similar activity comparable to the nalidixic acid reference drug (MIC 25 *μ*g/mL). Quantitative structural activity relationship and docking study of these compounds indicated that binding to the active site of bacterial penicillin-binding protein PBP-1b gives antibacterial activity to these synthesized derivatives [158].

Different mechanisms aim to explain the antibacterial action of flavone and isoflavone phytochemicals [139]. Flavone may form a complex with bacterial cell wall components preventing further adhesion. An example of this mechanism is the licoflavone **(18)** bioactive compound isolated from the flowers of the *Retama raetam,* which actively inhibits *E. coli* growth with MIC 7.81 *μ*g/mL [159]. Inhibition of bacterial enzymes is another mechanism as in artocarpin **(19)** compound extracted from *Artocarpus anisophyllus* Miq. leaves. This phytochemical exerts an antibacterial effect against MDR *E. coli* and *P. aeruginosa* [160]. Several studies in the synergistic effect of flavones with well-known antibiotics had been assessed, including the inhibition of bacterial efflux pumps, and increasing antibiotic susceptibility [139]. Baicalein **(20)** is a trihydroxyflavone present in the roots of *Scutellaria baicalensis* Georgi. and possesses various biological activities such as antiviral, antibacterial, anti-inflammatory, anticancer, and antioxidant properties [161]. Baicalein restores tetracycline and *β*-lactams activity against *MRSA,* and *E. coli* through the EPI mechanism [162]. In another study, the combination of baicalein with oxacillin and vancomycin were evaluated against *MRSA* and *VRE*. As a mechanism, this combination has a bactericidal effect via inhibition of bacterial cell wall synthesis. Time kill study revealed that the bacterial growth remarkably attenuated after 2–6 h [142].

Resveratrol **(21)** is a stilbene polyphenol that has recently gained extra attention for its benefits [163]. It has been studied for its antimicrobial potential alone and in combination with classical antibiotics [163]. Resveratrol exhibits moderate antibacterial activity toward Gram-positive *S. aureus* and *E. faecalis* (MIC 100–200 *μ*g/mL); however, it is less active against Gram-negative species such as *E. coli* and *K. pneumoniae* (MIC > 200 *μ*g/mL) [164]. As a mechanism, resveratrol inhibits biofilm production, and bacterial ATP synthase, thus decreasing the energy consumption required for bacterial growth. It also decreases bacterial toxin production, motility, and intervention with quorum sensing [163]. Eight bacterial strains, including MDR clinical isolates, were chosen to study resveratrol's in vitro antimicrobial activity [165]. According to the study, resveratrol displayed strong antimicrobial activity against *P. aeruginosa* and *S. aureus* (MIC 1.6 *μ*g/mL and 1.7 *μ*g/mL), respectively, compared to the norfloxacin reference drug (MIC 4.8 *μ*g/mL) [165]. In another study, resveratrol extracted from seeds of pinot noir grapes inhibited *Campylobacter jejuni* isolates' growth. Resveratrol acts as EPI for the CmeABC efflux pump responsible for the extrusion of antibiotics across the bacterial outer membrane [166].

Tannin is a water-soluble polyphenol commonly isolated from woody plants. It has been classified into two types: hydrolysable tannin, which is usually an ester of phenolic acid, and nonhydrolysable (condensed), a more abundant type of tannin usually derived from flavonoid dimers such as procyanidin [167]. Chebulinic acid **(22)** is hydrolysable tannin isolated from seeds of *Euphoria longana*, *Terminalia chebula* fruits, and leaves of *Terminalia macroptera* Guill. & Perr. Kunal patel et al. studied the binding affinity of 179 compounds to *Mycobacterium* DNA gyrase enzyme including chebulinic acid [168] which displayed a significant inhibitory effect on quinolone-resistant DNA gyrase. However, this study is based on virtual screening; further in vitro study is required to confirm this activity [168]. In another study, chebulinic acid extracted from *Terminalia chebula* showed potent antimicrobial action toward MDR *A. baumannii* [169].

Tannic acid **(23)**, another form of tannins, was proven to have efflux modulating properties against *MRSA* [170]. In a study, *MRSA* parent strains showed no efflux pump activity. However, with continuous use of fusidic acid, mutation emerges, and these mutant strains develop efflux pump type of resistance. It was shown that tannic acid administration with fusidic acid prevents mutation by acting as an alternative target for bacteria and promoting cell lysis [170]. Phenolic acids are natural compounds with one carboxylic acid functionality and many hydroxyl groups attached to the aromatic ring [171]. They could be derivatives of benzoic acid like gallic, protocatechuic, and *p*-hydroxybenzoic acids or cinnamic acids such as ferulic acid and *p*-coumaric acid [171]. Gallic acid **(24)** and ferulic acid **(25)** cause irreversible changes in *E. coli* and *P. aeruginosa* membranes through the reduction in negative surface charge and pore formation in cell membrane along with hydrophobicity changes, resulting in bacterial intracellular contents leakage [171].

A large-scale study was conducted to screen the antimicrobial effect of 239 traditional Chinese extracts against multidrug-resistant *S. aureus*. Some of these extracts reveal promising antibacterial activity (MIC ranging from 0.1 to 12.5 mg/mL and an MBC range of 0.78–25 mg/mL) as well as low cytotoxicity with median lethal concentration (LC_50_) >100 *μ*g/mL. The most active extracts include *Rhus chinensis* Mill., *Ilex rotunda* Thunb., *Leontice kiangnanensis* P.L.Chiu., *Oroxylum indicum* Vent., *Isatis tinctorial* L., *Terminalia chebula* Retz., *Acacia catechu* (L.f.) Willd., *Spatholobus suberectus* Dunn., *Rabdosia rubescens* (Hemsl.) H.Hara., *Salvia miltiorrhiza* Bunge., *Fraxinus fallax* Lingelsh., *Coptis chinensis* Franch., *Agrimonia Pilosa* Ledeb., and *Phellodendron chinense* C.K.Schneid. [172]. Further study is required to determine the active phytochemicals in these extracts and the potential mechanisms for their action.

### 3.3. Sulfur-Containing Compounds

**Figure 6:**
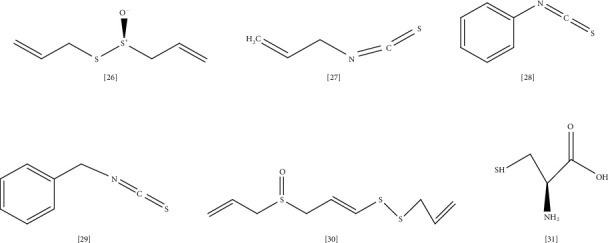
The chemical structures of selected sulfur-containing compounds reported with antimicrobial activity against MDR bacteria.

Volatile compounds with organo-sulfur structure have been proven to have broad antibacterial activity against Gram-positive and Gram-negative pathogens [173]. Little is known about their mechanism of action; however, the disulfide bond's presence confers a great role in their effectiveness [173]. Examples of these compounds are allicin, isothiocyanate, cysteines, and ajoene (Figure 6 lists the chemical structure of selected sulfur-containing compounds reported with antimicrobial activity). Garlic (*Allium sativum* L.) is a plant well recognized among other *Allium* species for its antimicrobial activity towards resistant bacteria [174]. This activity is related to garlic's organo-sulfur constituents, mainly allicin **(26)**, produced in response to garlic tissue damages and gives the crushed garlic its characteristic odor [174].

Several studies reported that allicin could inhibit MDR bacterial growth such as *S. aureus*, *E. coli*, *MRSA,* and *P. aeruginosa*; however, this effect is bacteriostatic, which suggested using allicin in combination with other antibiotics [175, 176]. As a mechanism, allicin inhibits bacterial enzymes like alcohol dehydrogenase, RNA polymerase, DNA gyrase, as well as inhibition of biofilm formation, DNA, and protein synthesis [175, 177]. Allicin presence in garlic was first reported by Cavallito and Bailey in 1944 [178]. It is a chemically unstable compound that rapidly decomposes to sulfide compounds such as ajoenes, diallyl sulfide (DAS), and diallyl disulfide (DADS) [175]. Thus, five analogues of allicin had been synthesized to overcome the stability problem [179]. The efficacy of these analogues was checked against bacteria and fungi, including *E. coli* and *Pseudomonas* spp. According to the study these analogues exhibited similar activity compared to allicin. The most potent antimicrobial activity was found in vapor form, concluding the use of allicin or its analogues in the gas phase as an alternative to antibiotics in the treatment of lung infection with MDR bacteria [179].

Plant family *Brassicaceae* represents several vegetables such as broccoli, mustard, cauliflowers, and cabbage. This family produces glucosinolates metabolites in response to tissue injury hydrolyzed by myrosinase enzyme into isothiocyanates (ITCs) [180]. Several natural as well as synthetic ITCs have been identified as potential antimicrobials against human pathogens, including resistant bacteria [181]. The antimicrobial activity of allyl isothiocyanate AITC **(27)** was isolated from a Japanese flower (*Wasabia japonica* (Miq.) Matsum.), and its synthetic analogue was evaluated against *E. coli* strain and *S. aureus* [182]. AITCs and their analogues sufficiently inhibit these bacteria's growth for up to 12 h. [182]. However, according to another study, this phenotype's efficacy is 100-fold less than polymyxin B antibiotic [183]. AITC affects bacterial membrane, similarly increasing cellular substance leakage to polymyxin B [183].

Another study on the AITC mechanism of action indicates that AITC may inhibit bacterial DNA synthesis by interfering with the sulfhydryl group in thioredoxin, promoting inferior enzyme activity, and interfering with the sulfhydryl group of acetate kinase, affecting energy metabolism of bacteria [184]. A previous study tested the effect of allyl isothiocyanate AITC, benzyl isothiocyanate BITC, and phenyl isothiocyanate PITC on *P. aeruginosa* [185]. These ITCS represent the major components of ITCs isolated from *Tropaeolum majus* L. and *Armoracia ruticana* plants. AITC, BITC, and PITC remarkably inhibited mature biofilm formation and showed synergy with meropenem by increasing its efficacy toward *P. aeruginosa* biofilm [185].

Phenyl isothiocyanate PITC **(28)** has proven to have moderate activity against *S. aureus* and *E. coli* (MIC 1000 *μ*g/mL) [186]. It modulates bacterial membrane changes through the interaction of electrophilic compounds with bacterial cells leading to decreased negative charges at the bacterial surface and increased surface hydrophilicity and electron donation. These changes trigger cellular disruption and loss of membrane integrity resulting in cell death. In addition, the synergism of PITC with ciprofloxacin and erythromycin was tested. The previous study recommended using PITC in combination with one of these two antibiotics as it enhances the antimicrobial activity of these antibiotics toward resistant *S. aureus* and *E. coli* [186]. ITCs isolated from *Cruciferous* species were tested for their antimicrobial effect against 15 *MRSA* clinical isolates from diabetic foot ulcers [187]. The antibacterial activity of ITCs mainly depends on their structural properties, with benzyl isothiocyanate BITC being the most active (bacteriocidal effect is up to 87%) [187].

As a mechanism, BITC structure has both lipophilic and electrophilic characteristics promoting disruption of bacterium ability to maintain membrane integrity through penetration of bacterial outer membrane [188]. Ajoene **(30)** is an organosulfur phenotype present in garlic extract and has two forms, E-ajoene and Z-ajoene [189]. This compound has broad pharmacological effects as an anticancer, antioxidant, antithrombosis, antiviral, antiparasitic and antimicrobial agent [190]. Although there is little knowledge about the mechanism of ajoene action, a study on ajoene antibacterial effect in *M. tuberculosis* suggested ajoene-induced stress response in the endoplasmic reticulum (ER), which represents part of the defense mechanism of the host against Mycobacterial infection [189].

Ajoene promotes reactive oxygen species (ROS) production through activation of c-Jun N-terminal kinase (JNK). Upon JNK activation, ROS production increased, resulting in the activation of ER stress and autophagy [189]. In another study, ajoene exhibited quorum sensor (QS) inhibiting activity, a system used by *P. aeruginosa* to synchronize for specific genes included in its pathogenicity [191]. A synthetic ajoene was used in both in vitro and in vivo models by the previous study. DNA microarray assay indicated that synthetic ajoene inhibited QS in a dose-dependent manner. Furthermore, it has biofilm killing property, which accounts for ajoene synergy with antibiotics such as tobramycin [191].

Antimicrobial peptides (AMPs) are a group of peptides that play a crucial role in the host defense mechanism [192]. AMPs occur in a variety of microorganisms, animals, and plant kingdoms [192]. With the emergence of antibiotic resistance, several strategies are proposed to tackle this problem, like using plant antimicrobial peptides from plants (PAMPs) as an alternative to antibiotics [193]. PAMPs are cysteine-rich cationic compounds **(31)** classified into six families according to cysteine number, amino acid chain, and disulfide bond contents: thionins, defensins, haveins, lipid transfer proteins, cyclotides, and snakins [194]. These PAMPs represent plant defense barriers against pathogens isolated from roots, flowers, seeds, stems, and leaves of several species [194]. In a recent study, PAMP was isolated from leaves of *Trianthema portulacastrum* L. and tested for antimicrobial activity toward MDR bacterial strains [193]. PAMP significantly inhibits the growth of *S. aureus* and *B. subtilis*, but it showed no activity toward *E. coli*. Further assessment is needed to identify the mechanism behind this action [193].

### 3.4. Terpenes

**Figure 7:**
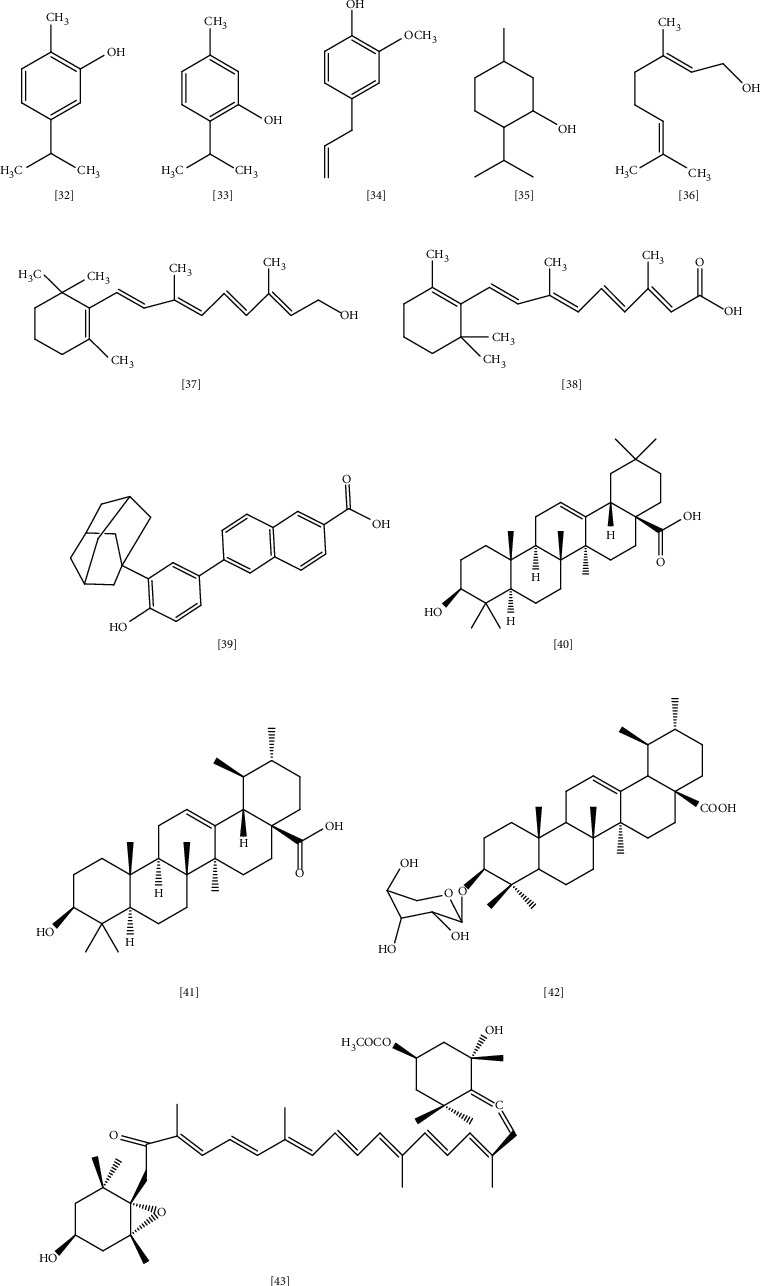
The chemical structures of selected terpenes reported with antimicrobial activity against MDR bacteria.

Terpenes are a large group of diverse organic molecules produced by various plants and animals, which are essential for their survival [195, 196]. Also, terpenes possess pharmacological and biological properties beneficial to humans. Chemically, terpenes are hydrocarbons with a 5-carbon isoprene unit as the main building block for their biosynthesis [197]. According to the number of isoprenes, terpenes are classified into monoterpenes (e.g., carvone, geraniol, and D-limonene), which account for 67% of biologically active terpenes [198], diterpenes (e.g., retinol and retinoic acid), triterpenes (e.g., betulinic acid and oleanolic acid), and tetraterpenes (e.g., *α*-carotene, *β*-carotene, and lutein) [199] (Figure 7 illustrates the chemical structures of selected terpenes with antimicrobial activity toward MDR bacteria).

Monoterpenes are volatile compounds representing the main constituents of plant's essential oil and are frequently used in fragrance and aromatherapy [199]. Several studies claimed the broad antimicrobial activity of carvacrol **(32)**, thymol **(33)**, eugenol **(34)**, and menthol **(35)** through efflux pump inhibition and inhibition of bacterial growth and membrane properties as well, with thymol being the most active toward *S. aureus* and *E. coli* [197, 200, 201]. Geraniol **(36)** exhibits significant activity against resistant Gram-negative pathogens such as *Enterobacter* species through efflux pump inhibition [202].

In another study, the effect of four monoterpenes on the biomembrane of *S. aureus* and *E. coli* were assessed through differential scanning calorimetry (DSC) technique [203]. This study tested the interaction between thymol, carvacrol, p-cymene, and gamma terpenes with dimyristoylphosphatidylcholine vehicle. The result indicated that thymol has a bactericidal effect on *S. aureus*. In contrast, carvacrol and p-cymene have an inhibitory action on *E. coli*. Both produce their effects in a concentration-dependent manner. Furthermore, the study suggested that disturbance of membrane lipid is the possible mechanism of action for these monoterpenes toward MDR pathogens, and the effect is highly dependent on lipid contents of the bacterial membrane [203].

Similarly, Garcia-salinas et al. conducted a study to demonstrate the effect of essential oils (EOs) from plants on the membrane properties of *S. aureus* and *E. coli* [204]. Carvacrol, cinnamaldehyde, and thymol were used in this study with chlorhexidine (0.004 mg/mL) as reference. SEM, flow cytometry, and confocal microscopy results indicated that these compounds effectively inhibit bacterial growth in vitro through disruption of membrane integrity. In addition, carvacrol, thymol, and cinnamaldehyde may affect bacterial biofilm formation in concentration equal or more than 0.5 mg/mL. It was suggested that the hydroxyl group in carvacrol and thymol has a role in this mechanism by inactivating microbial enzymes and interacting with the cell membrane. As a consequence, the leakage of intracellular components would be increased [204].

Retinol (vitamin A) **(37)** and its active form retinoic acid **(38)** are another class of terpenes present in carrots, sweet potatoes, pumpkin, squash, and cantaloupe [205]. Skin is the main target of these compounds, and several studies confirmed their activity in treating skin-related infectious diseases [206, 207]. As a mechanism, vitamin A contributes to gene expression through binding into two types of receptors—retinoic acid receptors (RAR) and retinoid X receptors (RXR)—which are abundant in the skin [208]. A new synthetic class of retinoid CD437 **(39)** was evaluated for potential bactericidal effect toward *E. faecalis* [209]. CD437 reported anti-biofilm activity toward this pathogen and profound synergy when combined with gentamycin [209]. In contrast, another study suggested that CD437 bacterial-killing property was linked to disruption of the lipid membrane [210]. In this study, two synthetic retinoids CD437 and CD1530 were tested for bactericidal action in *MRSA* and *S. aureus* infections.

Molecular dynamics simulations indicated that these compounds successfully penetrate and establish bacterial lipid bilayers. Moreover, these compounds reveal a significant synergistic effect with gentamycin against *MRSA* in mouse-model [210]. Triterpenes are a large, diverse group of phytochemicals with more than 20,000 compounds identified and recognized for broad pharmacological properties like anti-inflammatory, analgesic, cardiotonic, sedative, antimicrobial, and anticancer [211, 212].

Oleanolic acid (OA) **(40)** is triterpene isolated from 2000 plant species, mainly plants belonging to *Oleaceae* family such as the *Olea europaea* (olive), and functions as a barrier to protect the plant from water loss and pathogens [213]. Several studies have proven OA's efficacy and its derivatives as antimicrobial agents (MIC ≤ 100 *μ*g/mL) against *S. pneumoniae*, *E. faecalis*, *P. aeruginosa*, *M. tuberculosis*, *Streptococci*, and *E. coli* [211, 214–216]. Although there is little knowledge about OA action's mechanism, some studies proposed induction of stress response and efflux pump inhibition as primary targets for this class [211].

Ursolic acid (UA) **(41)**, which is another triterpenoid acid, shares most features in common with oleanolic acid (OA) [211]. It is abundant in the peel of berries, especially cranberries. Kurek et al. studied the mechanism of OA and UA action toward Gram-positive *Listeria monocytogenes* [217]. It was shown that both terpenoid acids reduce listeriolysin O hemolytic activity without influencing its synthesis or function. Besides, these compounds affect the biofilm formation of *Listeria monocytogenes*; however, the reason for such activity is still unclear and demands further clarification [217]. In another study, 3-O-*α*-L-arbinopyranoside (URS) **(42)**, an ursolic acid phenotype isolated from leaves of *Acanthopanax henryi* (Oliv.) Harms., was evaluated for its antimicrobial potential against *MRSA* alone and in combination with oxacillin [218]. MIC (6.25 *μ*g/mL), time-kill assay, white checkboard dilution test, and transmission electron microscopy (TEM) results indicated that URS has significant antimicrobial activity toward *MRSA* through inhibition of bacterial cell wall and induction of cell lysis mechanism. URS showed partial synergy with oxacillin, resulting in the suppression of the time-kill growth curve below the inhibitory level [218].

Carotenoids are natural pigments produced by several organisms such as bacteria, yeast, algae, molds, and plants with valuable physiological and pharmacological properties [219]. They acquired importance as a precursor for vitamin A as well as their potential as antioxidant and anticancer agents [220]. Carotenoids are classified into two groups: hydrocarbon carotenes (e.g., *β*-carotene, torulene) and oxygenated xanthophylls (e.g., astaxanthin, torularhodin) [220]. Few studies were performed to confirm the antimicrobial activity of carotenoids; among them is a study on fucoxanthin **(43)** carotenoids produced by algae [221]. According to the study, fucoxanthin showed promising antimicrobial effects toward Gram-positive and Gram-negative bacteria, including *S. aureus* (MIC 62.5 *μ*g/mL) [221]. However, further studies to demonstrate the possible mode of action is required.

Spathulenol, 1,8-cineole, trans-caryophyllene, *β*-pinene, *β*-eudesmol, camphor, *α*-pinene, and caryophyllene oxide are essential oils isolated from the leaves and flowers of *Salvia hydrangea* DC. ex Benth., an Iranian shrub that belongs to the *Lamiaceae* family. This plant has been used in traditional medicine to treat various bacterial and fungal infections. In the study, the isolated oils exhibited a significant inhibitory and lethal effect toward Gram-negative bacteria *P. aeruginosa* (MIC ∼ 16 *μ*g/mL) and *K. pneumoniae* (MIC ∼ 62 *μ*g/mL) [222]. These results highlighted the potential application of essential oils to develop new therapeutic agents in the future.

### 3.5. Coumarins

Coumarins are a group of natural products identified as secondary metabolites in plants, fungi, and bacteria with vast biological properties such as anti-inflammatory, anti-coagulant, antiviral, antibacterial, antifungal, vasodilation, anticancer, antihypertensive, antioxidant, and neuroprotective actions [223]. Coumarin is distributed to all parts of the plant, particularly in fruit (e.g., *Aegle marmelos* (L.) Corrêa., *Tetrapleura tetraptera* (Schum. & Thonn.) Taub.) [224], seeds (e.g., *Calophyllum cerasiferum* Vesque, *Calophyllum inophyllum* L.) [225], root (e.g., *Ferulago campestris* (Besser) Grecescu.) [226], and leaves (e.g., *Murraya paniculata* (L.) Jack.) [227].

**Figure 8:**
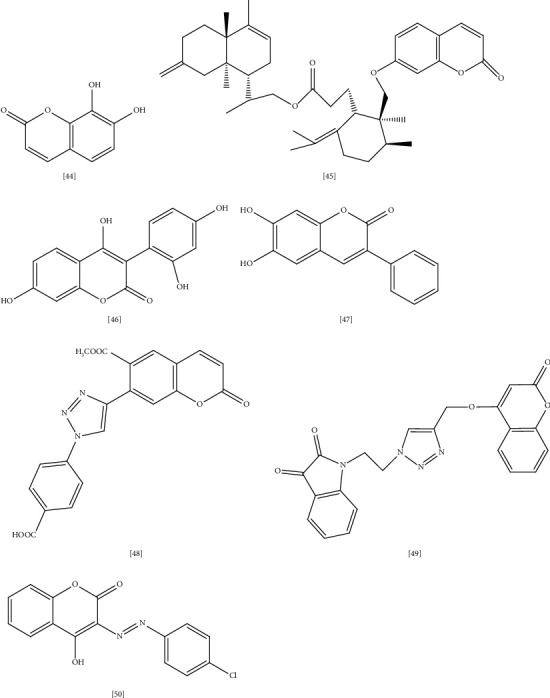
Selected coumarins with antimicrobial activity toward MDR bacteria.

Based on chemical structure, coumarins split into six subgroups: simple coumarins, furanocoumarins, dihydrofurano coumarins, pyrano coumarins (linear and angular types), phenyl coumarins, and bicoumarins [223]. Many studies manifested the antimicrobial activity of both natural and synthetic coumarins toward MDR bacteria. For instance, six coumarins isolated from the aerial part of *Rhododendron lepidotum* Wall. ex G. Don. exhibited considerable antimicrobial activity toward *MRSA* ATCC-15187, *E. coli* ATCC- 8739, *P. aeruginosa* ATCC-9027, with daphnetin **(44)** compound being the most active coumarin (MIC 125 *μ*g/mL) compared to ciprofloxacin reference drug [228] (Figure 8 illustrates selected coumarins with antimicrobial activity toward MDR bacteria).

In another study, disesquiterpene **(45)** coumarin obtained from the root of *Ferula pseudalliacea* Rech.f. showed higher antibacterial activity toward Gram-positive pathogens [229]. In this study, disesquiterpene activity was tested against *S. aureus* ATCC 25,922, *VRE, E. coli* ATCC25,922, *P. aeruginosa* PTCC1430, and *K. pneumoniae* in a concentration equal to 64 *μ*g/mL [229]. Moreover, asphodelin A **(46)**, which is an aryl coumarin from *Asphodelus microcarpus*, exhibited potent antimicrobial activity (MIC 4–128 *μ*g/mL) toward *S. aureus*, *E. coli*, and *P. aeruginosa*, whereas the glycoside form asphodelin A 4′-O-*β*-D-glycoside showed moderate activity [230]. Reports in the structural activity relationship (SAR) of coumarins suggested that lipophilic properties and planar structure are important for antibacterial effect as the mechanism of coumarin action involves cellular penetration through passive diffusion [231]. In addition, the SAR of three coumarins derived from *Streptomyces* species indicated that the sugar moiety of coumarin is essential for biological activity [232]. It is also suggested that inhibition of DNA gyrase enzyme is the mode of action of these coumarins [232].

Another study in the coumarin mechanism of action proposed that coumarin suppresses quorum-sensing activity, decreasing bacterial virulence and biofilm formation [233]. Previous reports indicated that oxidative stress plays a crucial role in antibiotic resistance [234, 235]. Thus, it is better to look for alternative medication with dual antibacterial and antioxidant activities. In accordance, a series of hydroxy-3-aryl coumarins were synthesized and screened for antibacterial and antioxidant activity toward MDR bacteria [236]. Docking studies were performed as well to determine the most active compound in this series. 3- phenyl coumarin with hydroxyl substitution at positions 5 and 7 **(47)** exhibited higher activity against *S. aureus* (MIC 11 *μ*g/mL), followed by *MRSA* (MIC 22 *μ*g/mL). Furthermore, a docking study suggested that tyrosyl-tRNA synthase, topoisomerase II, and DNA gyrase are the most appropriate binding sites in the bacterial target [236].

New series of coumarin triazole derivatives were synthesized and studied for in vitro antibacterial activity as well as binding properties to MurB protein [237]. It was noticed that substitution in position 6 or 7 of coumarin ring with 4-carboxyphenyltriazolyl **(48)** markedly enhance the antimicrobial activity toward *S. aureus* (MIC 0.16–6.28 *μ*g/mL) while bromine or aryl substitution in position 3 account for higher selectivity toward *E. coli* (MIC 0.02–0.15 *μ*g/mL) [237]. Similarly, other coumarin derivatives with indolinedione **(49)** were synthesized and studied for structural relationship on the antimicrobial activity against various Gram-positive and Gram-negative strains, including *E. coli* and *S. aureus* [238].

The SAR study established that the electronic environment of indolinedione positively affected the antimicrobial activity of these compounds, and the activity considerably reduced when the chain length between triazole and indolinedione portion increased. As a mechanism, docking studies suggested that binding to *S. aureus* dihydrofolate reductase is the mode of action of these hybrid molecules [238]. Some literature indicated that the metal complex formation between transition metals like cobalt, nickel, zinc, copper, and drug molecule significantly improves the drug's biological function [239, 240]. As an explanation, the metal complex serves as a vehicle for drug ligand, and the bond between the metal and ligand cleaves easily inside the body leading to accelerated drug action and increased efficacy [241, 242].

This approach is widely investigated to enhance antimicrobial, antifungal, and anticancer activities [243]. For instance, chromen-2-one complex of 3-aryl-azo-4-hydroxy coumarin **(50)** exhibited excellent antimicrobial activity toward *E. coli* (MTCC 614), *K. pneumoniae* (MTCC 109), and *S. aureus* with MIC equal to 31.25 *μ*g/mL [240]. In a recent study, a new coumarin compound 3,3′-(3,4-dichlorobenzylidene)-bis-(4-hydroxycoumarin) was detected as an inhibitor to *MRSA* biofilm formation. Molecular docking study suggested that the anti-*MRSA* effect is due to targeting bacterial arginine repressor (ArgR), a property that makes this compound a promising source for the development of new anti-*MRSA* agents [244, 245].

## 4. Conclusions and Future Perspectives

Nature represents an abundant source for bioactive compounds that are available, affordable, and easy to isolate with almost no harm to humans. Plant-derived compounds have been used since ancient times to treat various infectious diseases. For instance, honey is used as an ointment since the medieval era to treat wounds and prevent secondary infections. Also, *Echinacea* extract was used by native Americans for the same purpose. With the rapid progression of bacterial resistance to conventional antibiotics, scientists returned to nature, searching for alternatives. Many studies had been done, and the therapeutic potential of plant-derived compounds has been documented. Some of these compounds are active as antibiotics when they are used alone while others may enhance the antibacterial action when coadministered with existing antibiotics. Thus, combination therapy of classical antibiotics with phytochemicals could be a promising solution to overcome resistance. However, designing new medication from the plant is still challenging.

Various mechanisms have been proposed to explain plant-derived compounds' action, including efflux pump inhibition, destruction of the bacterial membrane, inhibition of biofilm formation, DNA, and protein synthesis. Understanding the specific mechanism behind phytochemical action is vital to develop a new antimicrobial agent. Nevertheless, shifting from in vitro study to in vivo study and clinical trial remains a big confrontation since several factors may affect the efficacy of phytochemicals like tissue penetration and bioavailability. As such, a better understanding of pharmacokinetic and pharmacodynamic of the natural molecule is necessary.

## Figures and Tables

**Table 1:** Mechanism of action and antibiotics affected by common MDR bacteria.

MDR type	WHO priority category	Mechanism of resistance	ABs class	Reference
*Pseudomonas aeruginosa*	Critical	Efflux pump (MexAB-OprM)	*β*-Lactams and penem groups of ABs	[60]
		Enzyme inactivation (*β*-lactamase)		
		Alteration of membrane permeability		

*Acinetobacter baumannii*	Critical	Enzyme inactivation (aminoglycoside-modifying enzyme)	*β*-LactamsAminoglycosides	[61]
		Efflux pump		
		Change membrane permeability		

*Klebsiella pneumoniae*	Medium	Alteration of target site	3^rd^ generation cephalosporins *β*-Lactams Carbapenem	[62]
		Enzyme inactivation (*β*-lactamase)		
		Efflux pump		
		Alteration of membrane permeability		

*MRSA, VRSA, VISA*	High	Binding site alteration Mutation in genes involved in cell wall synthesisEfflux pump (NorA)	Methicillin	[7]
			Oxacillin	
			Vancomycin	
			Penicillin	
			Cephalosporins	
			Carbapenem	

*VRE*	High	Alteration of target site	Most ABs	[63]
			Vancomycin	
			Daptomycin	
			Linezolid	

*CRE*	Critical	Enzyme inactivation(*β*-lactamase and carbapenemase)	*β*-Lactams	[64]
			Most ABs	
			Carbapenem	

*Escherichia coli*	Critical	Efflux pump (AcrAB-TolC)	Trimethoprim, amoxicillin, gentamycin, tetracycline	[65]

*Helicobacter pylori*	High	Mutation in the domain V of 23S rRNA gene of the bacteria	Clarithromycin	[46]

MRSA: methicillin-resistant staphylococcus aureus, VRSA: vancomycin-resistant staphylococcus aureus, VISA: vancomycin intermediate staphylococcus aureus, VRE: vancomycin-resistant enterococci, and CRE: carbapenem-resistant *Enterobacteriaceae*.

**Table 2:** Plants reported with antimicrobial activity against MDR bacteria from 2015to 2021.

Plant	Part	Active compound	Mechanism of action	Active against^*∗*^	Findings	Reference
*Alkanna tinctoria*	Leaves	Alkaloids		*MRSA, E. coli, P. aeruginosa, A. baumannii*	MIC well diffusion methodMBC model	[73]
		Flavonoids				
		Carbohydrates				

*Rhazya stricta* Decne.	Leaves	Alkaloids	Cell membrane disruption	*MRSA, E. coli, K. pneumoniae, VRE*	TEM analysis	[74]

*Holarrhena antidysenterica*		Conessine alkaloid	Efflux pump inhibition	*P. aeruginosa*	MIC model	[75]
					NPN uptake assay	
					Active against RND family	

*Allium sativum* L. (garlic)	Fruit	Allicin (sulfur-containing compound)		*P. aeroginosa*	MIC and MBC models	[76]
					In vivo	

*Oxalis corniculata*	Leaves			*MDR Salmonella typhi*	MIC and MBC well diffusion methods	[18]
				*K. pneumoniae*		

*Coula edulis* Baill.	Fruit	Alkaloids, flavonoids, saponin	Efflux pump inhibition	*E. coli*	MIC and MBC models	[77]
		Cardiac glycosides		*K. pneumoniae*		

*Mangifera indica* L.	Bark	Carotenoid	Efflux pump inhibition	*P. aeruginosa*	MIC and MBC models	[77]
		Tannin				
		Catechin				
		Polyphenol				

*Citrus sinensis*	Peel	Polyphenol	Efflux pump inhibition	*E. coli*	MIC and MBC models	[77]
		Catechin				
		Carbohydrates				

*Moringa oleifera* Lam.	Leaves	Alkaloids,		*P. aeruginosaKlebsiella spp.E. coli*	MIC and MBC models	[78]
		Polyphenols				
		Flavonoids				
		Anthraquinones				
		Coumarin				
		Tannin, saponin				
		Terpenes, sterols				

*Matricaria recutita* L.	Flowers			*P. aeruginosa*	MIC and MBC model	[78]
				*Klebsiella spp.*		
				*E. coli*		

*Eleutherine bulbosa* (Mill.) Urb.	Bulb extract			*S. aureusShigella boydii*	MIC and MBC model	[79]
					Time kill study	
					TLC-bioautography	

*Zanthoxylum alatum*	Leaves, stem	Fenchol, linalool		*E. coliK. pneumoniae*	In vitro model	[80]
					MIC	

*Cinnamomum tamala*	Leaves	Cinnamaldehyde		*MDR-H. pylori*	In vitro model	[80]

*Ocimum sanctum* L.	Leaves			*S. aureus-*resistant strains	In vitro model	[80]
					MIC	

*Zanthoxylum armatum* DC.	Fruit			*E. faecium*	MIC	[81]
				*S. aureus*	Biofilm and quorum sensitivity assay	
				*K. pneumoniae*	*δ*-Toxin inhibition	
				*A. baumannii*	Mammalian cytotoxicity study	
				*P. aeruginosa*		

*Adiantum capillus-veneris* L.	Whole plant			*E. faecium*	MIC	[82]
				*S. aureus*	Biofilm and quorum sensitivity assay	
				*K. pneumoniae*	*δ*-Toxin inhibition	
				*A. baumannii*	Mammalian cytotoxicity study	
				*P. aeruginosa*		

*Artemisia absinthium* L.	Aerial parts			*E. faecium*	MIC	[82]
				*S. aureus*	Biofilm and quorum sensitivity assay	
				*K. pneumoniae*	*δ*-Toxin inhibition	
				*A. baumannii*	Mammalian cytotoxicity study	
				*P. aeruginosa*		

*Martynia annua* L.	Fruit			*E. faecium*	MIC	[82]
				*S. aureus*	Biofilm and quorum sensitivity assay	
				*K. pneumoniae*	*δ*-Toxin inhibition	
				*A. baumannii*	Mammalian cytotoxicity study	
				*P. aeruginosa*		

*Cynodon dactylon* (L.) Pers.	Whole plant			*MRSA*	MIC and MBC models	[82]
				Imipenem-resistant *P. aeruginosa*		
				*MDR- salmonella typhi*		

*Ocimum basilicum* L.		Phytol, cadinene		*A. baumannii*	MIC using broth microdilution technique	[83]
				*E. coli*		

*Plectranthus barbatus* Andrews.		Phytol, camphor, verbenone		*A. baumannii*	MIC using broth microdilution technique	[83]
				*K. pneumoniae*		
				*E. coli*		
				*P. aeruginosa*		

*Rosmarinus officinalis* L.		Phytol, camphor, verbenone		*A. baumannii*	MIC using broth microdilution technique	[83]
				*K. pneumoniae*		

*Myrtus communis* L.	Seeds	Gallic acid		*S. aureus*	MIC model	[60]
		Ellagic acid		*P. aeruginosa*		
		Flavonoids		*E. coli*		
		Fatty acid, tannin		*S. enteric*		

*Cinnamomum zeylanicum*	Leaves	Polyphenol		*S. aureus*	MIC model	[60]
				*P. aeruginosa*		
				*E. coli*		
				*S. enteric*		

*Syzygium legatii* Burtt Davy & Greenway.	Leaves			*E. coli*	MICIn vitro toxicity study using Caco-2 cells	[84]
	
*Eugenia zeyheri* (Harv.) Harv.						

*Peganum harmala* L.	Seeds	Alkaloids		*MRSA*	MICMTT assay using HEK-293 cells	[85]
		Harman, harmine				
		Harmaline, harmalol				

*Glycyrrhiza glabra* L.	Fruit & leaves	Alkaloids, saponin		*P. aeruginosa*	MIC model	[86]
		Tannin, flavonoids				
		Phenols, coumarin, terpenes				

*Ficus sycomorus* L.	Leaves	Flavonoids		*A. baumannii*	MIC and MBC models	[87]
		Phenols		Resistant *S. aureus*		
*Syzigium cumini*	Leaves	Alkaloids		*MRSAE. coli*	MIC and MBC models	[88]
		Flavonoids				
		Terpenoids				

*Punica granatum* L.	Peel	Ellagic tannin		*P. aeruginosa*	MIC and MBC models	[89]
		Ellagic acid				
		Gallic acid				

*Camellia sinensis* (green tea)	Leaves			*MRSA*	MIC model	[90]

*Mentha longifolia* (L.) L.	Arial part			*VRE*	MIC model	[90]

*Croton macrostachyus* hochst. ex Delile.	Leaves	Triterpenes		*MRSA*	MIC and MBCBroth microdilution method	[91]
		Sterols, polyphenols				
		Saponins				

*Catharanthus roseus* (L.) G.Don.	Leaves	Alkaloids, triterpenes		*MRSA*	MIC and MBCBroth microdilution method	[91]
		Sterols, flavonoids				
		Polyphenols				

*Paullinia pinnata* L.	Leaves	Triterpenes		*MRSA*	MIC and MBCBroth microdilution method	[91]
		Sterols, polyphenols				
		Saponins				

*Anacardium occidentale* L.	Leaves	Alkaloids, saponin		*E. coliK. pneumoniae*	MIC agar well diffusion method	[92]
		Flavonoids, tannin				
		Phenol anthocyanin				

*Thymbra spicata* L.	Arial parts	Carvacrol, thymol		*E. coli*	MIC and MBC microdilution method	[93]
		Camphor				

*Lawsonia inermis* (henna)	Leaves	Alkaloids, terpenoids, phenolic compounds, tannins, steroids, anthraquinones		*MRSA* ATCC43300	MIC and MBC (agar well diffusion and colorimetric microdilution methods)	[94]
				*K. pneumoniae*		
				ATCC700603		
				*P. aeruginosa* ATCC37853		

*Azadirachta indica* (neem)	Leaves	Alkaloids, terpenoids, phenolic compounds, tannins, steroids, saponins, flavonoids		*MRSA* ATCC43300	MIC and MBC (agar well diffusion and colorimetric microdilution methods)	[94]
				*K. pneumoniae*		
				ATCC700603		
				*P. aeruginosa* ATCC37853		

*Piper betle* Linn.	Leaves			*P. aeruginosa* TISTR1287	Agar-disc diffusion method	[95]
					Broth dilution assay (MIC and MBC)	

*Cistus salviifolius*		Hydrolysable tannins, flavonoids (myricetin and quercetin)		*MRSA*	Disc-dilution method, microdilution method	[96]

*Punica granatum*		Hydrolysable tannins (punicalin and punicalagin)		*MRSA*	Disc-dilution method, microdilution method	[96]

*Platanus hybrida*	Fruits	Phenolic compounds		*E. faecium, E. faecalisP. aeruginosa, K. pneumoniae*	Kirby–Bauer disc diffusion method	[97]

*Syzygium aromaticum*	Flower buds			*K. pneumoniaeS. aureus*	Disc-diffusion method (MIC and MBC), scanning electron microscopy, DNA apoptosis	[98]

*Acacia nilotica*	Seeds			*S. aureus*	Disc-diffusion method (MIC and MBC), scanning electron microscopy, DNA apoptosis	[98]

^*∗*^All bacteria strains are multidrug resistant.

**Table 3:** Chemical structures of alkaloidal compounds with antimicrobial activity against MDR bacteria.

Chemical structure	Active against	Ref.
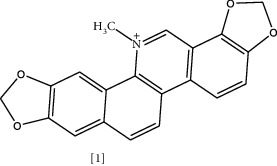	*MRSA, E. coli, S. aureus*	[104–106]
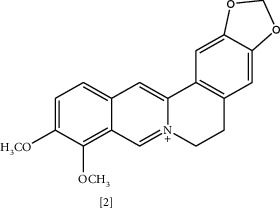	*E. coli, MRSA,* synergistic action against *Staphylococcus* spp.	[25, 107, 108]
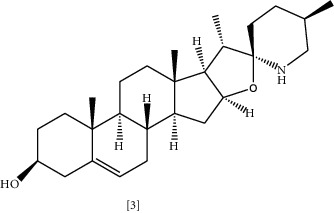	*MRSA*	[109]
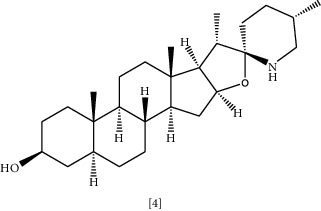	*S. aureus, E. faeclis, P. aeruginosa, E. coli*	[110]
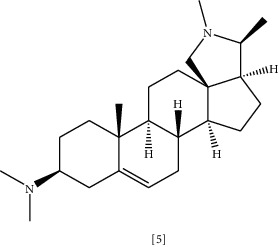	*P. aeruginosaA. baumannii*	[111, 112]
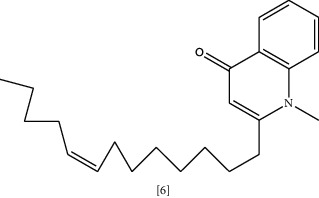	*M. tuberculosis*	[113]
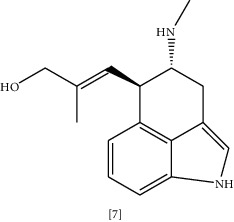	*E. coli*	[114]

## Data Availability

No data were used to support this study.

## Abbreviations

MDR: Multidrug resistance
EP: Efflux pump
*E. coli*: *Escherichia coli*
*MRSA*: Methicillin-resistant *Staphylococcus aureus*
*K. pneumoniae*: *Klebsiella pneumoniae*
*P. aeruginosa*: *Pseudomonas aeruginosa*
*VRE*: Vancomycin-resistant *Enterococci*
*VRSA*: Vancomycin-resistant *Staphylococcus aureus*
*VISA*: Vancomycin-intermediate *Staphylococcus aureus*
*CRE*: Carbapenem-resistant *Enterobacteriaceae*
MIC: Minimum inhibitory concentration
RND: Resistance-nodulation family
SMR: Small multidrug resistance
MFS: Major facilitator superfamily
MATE: Multidrug and toxin extrusion
ABC: Adenosine triphosphate binding cassette.
